# Microfluidic Chromatography for Enhanced Amino Acid Detection at Ocean Worlds

**DOI:** 10.1089/ast.2021.0182

**Published:** 2022-09-05

**Authors:** Tessa Van Volkenburg, Jennifer Skerritt Benzing, Kathleen L. Craft, Korine Ohiri, Ashley Kilhefner, Kristen Irons, Christopher Bradburne

**Affiliations:** ^1^ Johns Hopkins University Applied Physics Laboratory, Laurel, Maryland, USA.; ^2^ University of North Carolina at Chapel Hill College of Arts and Sciences, Chapel Hill, North Carolina, USA.

**Keywords:** Amino acid, Salt, Cation exchange chromatography, Ocean world, Desalination, Purification

## Abstract

Increasing interest in the detection of biogenic signatures, such as amino acids, on icy moons and bodies within our solar system has led to the development of compact *in situ* instruments. Given the expected dilute biosignatures and high salinities of these extreme environments, purification of icy samples before analysis enables increased detection sensitivity. Herein, we outline a novel compact cation exchange method to desalinate proteinogenic amino acids in solution, independent of the type and concentration of salts in the sample. Using a modular microfluidic device, initial experiments explored operational limits of binding capacity with phenylalanine and three model cations, Na^+^, Mg^2+^, and Ca^2+^. Phenylalanine recovery (94–17%) with reduced conductivity (30–200 times) was seen at high salt-to-amino-acid ratios between 25:1 and 500:1. Later experiments tested competition between mixtures of 17 amino acids and other chemistries present in a terrestrial ocean sample. Recoveries ranged from 11% to 85% depending on side chain chemistry and cation competition, with concentration shown for select high affinity amino acids. This work outlines a nondestructive amino acid purification device capable of coupling to multiple downstream analytical techniques for improved characterization of icy samples at remote ocean worlds.

## 1. Introduction

The detection of life outside Earth would be an incredible discovery, revolutionizing our understanding of life and providing insight into how it develops and persists in various environments. The Planetary Science Decadal Survey for 2013–2022 emphasizes the development of capabilities to enable the search for extraterrestrial life, including explorations into where organic synthesis occurs today, modern habitats containing necessary habitable conditions, and sampling environments for organisms possibly living there now (National Research Council, 2011). In that regard, some outer solar system ocean worlds may have conditions conducive to harboring life, including Jupiter's icy moons Ganymede, Callisto, and Europa (Greenberg *et al.,* 2000; Chyba and Phillips, 2001), the latter of which likely contains a global ocean beneath its icy surface (Carr *et al.,* 1998; Iess *et al.,* 2014). Potential ocean worlds also orbit Saturn, which include Rhea (*e.g.,* Hussmann *et al.,* 2006), Titan, and Enceladus. Enceladus has confirmed water either as a global ocean (Nimmo and Pappalardo, 2016; Thomas *et al.,* 2016; Patthoff *et al.,* 2019) or local sea (Collins and Goodman, 2007) that is the source for geyser activity observed at the South Polar Terrain (Porco *et al.,* 2006). Neptune's Triton may also have water with observed plume activity (*e.g.,* Soderblom *et al.,* 1990). For Europa and Enceladus, the moons' eccentric orbits around their respective planets cause frictional heating within the interior that may provide the energy needed to maintain their oceans and chemical elements at the seafloor, making each a likely candidate for supporting a habitable environment (*e.g.,* Reynolds *et al.,* 1983; Greenberg, 2005).

A robust strategy in the search for life on one of these ocean worlds would employ various techniques to detect several biosignatures (*e.g.,* Hand *et al.,* 2017; MacKenzie *et al.,* 2021). Multiple detections or nondetections of multiple biosignature types would increase confidence in life detection, or lack thereof. Two important biosignatures are the abundance distribution and chirality of amino acids. The Ladder of Life Detection categorizes amino acids as potential biomolecule components, and their high detection sensitivity and stability in harsh conditions compared to larger biopolymers (*i.e.,* DNA and RNA) make them desirable targets (Neveu *et al.,* 2018). However, since amino acids can form both biotically and abiotically, instruments developed must couple detection with studies into the chirality and abundance of amino acids in the mixture (Creamer *et al.,* 2017). On Earth, proteinogenic amino acids appear almost exclusively in the l-configuration, which is essential for preserving catalytic capabilities of protein folding and resisting nucleic acid mutations. Previous abiotic meteorite studies have found mainly racemic mixtures of amino acids, or ratios of l- to d-configured amino acids with less than a 20% enantiomeric excess (Cronin and Pizzarello, 1997; Glavin and Dworkin, 2009). This indicates that large homochiral distributions that favor either configuration (>20%) would suggest evidence of life. Additionally, the type of amino acid and its relative abundance in the mixture can also indicate life. Though there is much discussion on how to identify likely biotic precursors, previous research has hypothesized looking for large ratios of thermodynamically unfavorable amino acids (ones with high energy costs to manufacture biosynthetically) or amino acids with high catalytic propensity (histidine, cysteine, and arginine) (Patel *et al.,* 2015; Georgiou, 2018). Regardless of the method, preserving amino acid ratios in the sample during analysis is paramount to proving biogenicity.

A considerable challenge to remote amino acid detection is the expected difficulty in analyzing *in situ* samples. Samples at ocean worlds will likely have complex compositions and low biosignature concentrations. At Enceladus, apart from water (H_2_O), the Cassini spacecraft detected possible molecular nitrogen (N_2_), carbon dioxide (CO_2_), methane (CH_4_), propane (C_3_H_8_), acetylene (C_2_H_2_), and several other species in the plume material (Waite *et al.,* 2017; Postberg *et al.,* 2018) and has observed carbon oxides (CO, CO_2_) and a small amount of ammonia (NH_3_) on the surface (Brown *et al.,* 2006; Hendrix *et al.,* 2010). At Europa, observations of the surface indicate the presence of salts and other non-ice materials, including sodium and magnesium chlorides (NaCl, MgCl_2_), magnesium sulfate (MgSO_4_), sulfur from Jupiter's moon Io, hydrogen peroxide (H_2_O_2_), and possibly radiation-processed sulfuric acid (H_2_SO_4_) (McCord *et al.,* 1999; Kargel *et al.,* 2000; Dalton, 2007; Carlson *et al.,* 2009; Brown and Hand, 2013). In particular, salt-rich environments are of great scientific interest to astrobiologists, as such places harbor biology on Earth (Mikucki *et al.,* 2016; Perl and Baxter, 2020). Yet high ratios of salts can inhibit biosignature analysis techniques by degrading signal resolution (*e.g.,* Stockton *et al.,* 2009) or reducing derivatization efficiencies (*e.g.,* Gehrke and Leimer, 1970). In addition to complex chemistries, the expected low energy supplied by tidal heating at these worlds indicates that any biomarkers present may be more dilute than Earth's ocean. Terrestrially, metagenomics sampling campaigns have reported biomass loading ranging from parts per million (ppm) to parts per trillion (ppt) concentrations depending on the sampling location, depth, and time of year (Biller *et al.,* 2018). These low biomarker concentrations on Earth, a place teeming with life, necessitate the development of sensitive instruments that function independently of the sample chemistry introduced or that include devices with adequate preparation steps to purify and concentrate the sample.

### 1.1. Salt interference with amino acid detection

In terms of detection, mass spectrometry (MS) is the gold standard for forensic analysis to determine not only chemical composition but also structure by using fragmentation patterns. To detect amino acids, it is necessary to use a separation technique ahead of downstream analysis to identify distinct molecular peaks, such as gas chromatography (GC), high-performance liquid chromatography (HPLC), or capillary electrophoresis electrospray ionization (CESI) (*e.g.,* Hušek and Macek, 1975; Abrahamsson *et al.,* 2021; Zamuruyev *et al.,* 2021). Historically, *in situ* spacecraft landers have utilized reversed-phase GC columns, including the Cometary Sampling and Composition (COSAC) experiment on the European Space Agency's Rosetta Philae lander that sampled cometary material of 67P/Churyumov-Gerasimenko (Thiemann and Meierhenrich, 2001; Altwegg *et al.,* 2017; Boehnhardt *et al.,* 2017). On Mars, both the Sample Analysis at Mars (SAM) suite currently on the Curiosity rover and the Mars Organic Molecule Analyser (MOMA) planned for the 2022 ExoMars Rosalind lander utilize GC-MS detection (Mahaffy *et al.,* 2012; Goesmann *et al.,* 2017; Guzman *et al.,* 2020).

For GC-MS, derivatization is a critical step to make amino acids volatile and thermally stable; however, salt content or an extreme pH in the sample can inhibit the reaction (Gehrke and Leimer, 1970). During laboratory testing for the SAM instrument suite with Mars soil analogs, Stalport *et al.* (2012) found the derivatization yield of amino acids with DMF-DMA (N,N-dimethylformamide dimethyl acetal) and MTBSTFA-DMF (N-methyl-N-(tert-butyldimethylsilyl) trifluoroacetamide and dimethylformamide) depends strongly on the mineral matrix. The presence of hydrated minerals or oxides in martian soil impedes derivatization, necessitating extraction with polar solvents such as DMF and isopropyl alcohol to achieve higher yields (Buch *et al.,* 2006). In addition, accumulation of nonvolatile salts onto GC columns will reduce signal and impede consistent analyses (Gehrke and Leimer, 1970; Rood, 2007) or form adducts in MS, which can lower signal and sensitivity (Zhao *et al.,* 2002). Historically, acid hydrolysis was used to extract amino acids from regolith matrices. This entails soaking the sample in hot water, hydrolyzing a fraction with acid, and desalting on a cation exchange column (Botta and Bada, 2002). More recent work has developed a subcritical water extraction method, which heats soil samples in high-pressure liquid water (Amashukeli *et al.,* 2007; Sephton and Botta, 2008; Noell *et al.,* 2018) and can be made modular for remote deployment (Kehl *et al.,* 2019; Mora *et al.,* 2020). Subcritical water extraction causes water to exhibit a lower dielectric constant, weakening the hydrogen bonds and allowing similar extraction efficiencies of organics to less polar solvents such as methanol and ethanol (Ko *et al.,* 2020). Noell *et al.* (2018) also demonstrated that adding dilute hydrochloric acid reduced the time and temperature needed for extraction without decreasing efficiencies but required subsequent desalting before HPLC analysis. Alternatively, the recent MOMA module utilizes laser desorption to volatilize samples, which has been shown to be less sensitive to oxidizing reagents (Guzman *et al.,* 2020) but is limited to surface chemistries and requires a solid matrix to assist liquid sample desorption (matrix-assisted laser desorption/ionization, MALDI). These studies show that, to improve amino acid detection, sensitivity, and resolution with unknown sample matrices, an upstream sample desalination step is necessary before derivatization.

For amino acid detection at ocean worlds, like on Mars, understanding the chemical context of the sample matrix is essential for sensitive biosignature detection (Willis *et al.,* 2021). To that end, researchers are developing techniques and instruments that include monolithic automatic sample processing systems capable of small volume fluid routing; dehydrated reagent storage; and pressure, pH, redox potential, and conductivity measurements (Chinn *et al.,* 2017; Noell *et al.,* 2019; Radosevich *et al.,* 2019). Compared to traditional large-scale chromatography, microfluidic sample preparation of amino acids on Earth is an attractive option due to the low cost, small input volumes needed, and rapid processing times (Ou *et al.,* 2020). Microfluidic devices' lightweight construction and small form factor make them ideal modules for space applications as well (Hessel *et al.,* 2020). Typically, electrical or chemical methods are utilized for microfluidic systems rather than size exclusion, due to the high pressures (>20 bar) needed for amino acid separation (Biswas *et al.,* 2018). Previous research into microfluidic *in situ* spacecraft landers has focused on capillary electrophoresis and shown separation and detection of a variety of biologically relevant molecules including amino acids, aldehydes and ketones, carboxylic acids, and thiols (Skelley *et al.,* 2005, 2006; Chiesl *et al.,* 2009; Benhabib *et al.,* 2010; Stockton *et al.,* 2010, 2011; Mora *et al.,* 2011, 2013; Jensen *et al.,* 2013; Kim *et al.,* 2013; Creamer *et al.,* 2017). This powerful separation modality can achieve lower levels of detection than MS when paired with laser-induced fluorescence (LIF) detection (Willis *et al.,* 2021), with a recent study by Creamer *et al.* (2017) demonstrating simultaneous chiral separation of 17 amino acids using capillary electrophoresis laser-induced fluorescence (CE-LIF) at detection levels of 5 nM for neutral amino acids and 500 nM for acidic amino acids. More recently, Mora *et al.* (2020) also demonstrated nanomolar sensitivities in an autonomous sample-to-analysis platform dubbed the “Chemical Laptop” deployed in the Atacama Desert, Chile. However, many fluorescent tags used in capillary electrophoresis are susceptible to highly acidic or salty samples, particularly multivalent cations, which hinder sensitive detection. Strong acids interfere with the labeling reaction, and concentrated salts cause broader detection peaks with lower resolution. In extreme cases, concentrated salts disrupt the electro-osmotic flow in the channels. While higher ionic strength buffers and chelating agents neutralize these effects, this mitigation step requires knowledge of the initial sample composition (Stockton *et al.,* 2009). To circumvent this, other work has focused on capillary electrophoresis with capacitively coupled contactless conductivity detection (CE-C^4^D), which allows simultaneous resolution of both cations and biomolecules of interest like amino acids (Ferreira Santos *et al.,* 2018) and carboxylic acids (Jaramillo *et al.,* 2021). This method reduces the amount of buffers needed for analysis; however, it provides lower detection sensitivity.

### 1.2. Cation exchange for amino acid desalination

A way to purify unknown mixtures of amino acids in solution is through liquid chromatography ion exchange resins. In these methods, a charged resin binds the amino acids, and subsequent buffers remove contaminants before eluting purified amino acids. Appropriate resin and buffer selection is paramount to ensuring high recovery yields of diverse amino acids without affecting downstream derivatization and analysis. Cation exchange is the most commonly used ion exchange chromatography resin and has previously been shown to desalt planetary relevant samples, including meteorites, lunar regolith, and martian analog soil samples (*e.g.,* Zhao and Bada, 1995; Simkus *et al.,* 2019; Glavin *et al.,* 2020). The fundamental chemistry is well established; affinity between the positively charged amine groups on the amino acids bind to the negatively charged sulfonic acid functional groups on the resin. However, many variables affect the efficacy of this phenomenon, including, but not limited to, pH of the buffering solutions and sample introduced, residence time of buffers on the column, and ambient temperature of the system (Wang *et al.,* 1989; Melis *et al.,* 1996).

Recent work has found on average a 2.5 ppb limit of detection for mixtures of amino acids on a cation-exchange chromatography resin coupled with electrospray ionization mass spectroscopy (ESI-MS) (Abrahamsson *et al.,* 2021). Of particular interest is the Bio-Rad Laboratories strong cation exchange resin (AG50), which utilizes a styrene divinylbenzene copolymer lattice with sulfonic acid functional groups. Previous research has confirmed a high recovery efficiency of 94.3 ± 11.4% (*n* = 3) for both proteinogenic and abiotic amino acids when using this technique (Takano *et al.,* 2010). Thus, evidence exists that cation exchange achieves both high sensitivity and high recovery. A brief summary of this and other such work is included in Table S1. This technique is widely used in laboratory studies; however, to date and to our knowledge, there are no published results of this resin integrated into a dedicated microfluidic system or investigations into the effect of ocean sample composition on the separation kinetics and amino acid recovery.

The work presented herein outlines a simple and lightweight microfluidic chromatographic device capable of desalting astrobiologically relevant samples to enable high-resolution downstream detection. During development, we investigated amino acid capture efficiency and salt removal with three different europan analog salts at high salt-to-amino-acid molar ratios. Additionally, we tested competition between mixtures of proteinogenic amino acids under challenging conditions, including concentrations close to the downstream detection limit and purification in a terrestrial ocean sample. In the latter, we demonstrated concentration of some high-affinity and biologically relevant amino acids. Coupled with downstream analyses instrumentation, application of this technique could enable *in situ* detection of amino acids on ocean worlds, even from complex, low amino acid concentration samples, and provide an indication of previous or extant life.

## 2. Materials and Methods

### 2.1. Buffer and resin materials

This project employed biotechnology grade AG50W-X8 cation exchange resin with Hydrogen form, 8% crosslink density, 200–400 dry mesh size, 63–150 μm wet bead size, and ∼1000 MW limit (purchased from Bio-Rad Laboratories). Deionized (DI) water was obtained from a Milli-Q water purification system (Millipore Corp.) or from NERL reagent-grade water (Fisher Scientific). Both sources were rated at 18 MΩ/cm specific resistance and filtered to 0.2 μm. ACS-grade reagents (Millipore Sigma), hydrochloric acid (HCl, 37 w/v %), and ammonium hydroxide (NH_4_OH, 28–30 w/v %) were diluted in DI water to 10 mM and 1.0 M, respectively. All buffers were prepared in biosafety hoods to reduce contamination.

### 2.2. Microfluidic chip fabrication and testing

Early prototypes of the cation exchange module were fabricated by using conventional soft lithographic procedures for polydimethylsiloxane (PDMS) based microfluidic chips (Qin *et al.,* 2010). Briefly, a 300 μm tall SU-8 based master mold was fabricated using photolithography. The design consisted of inlet/outlet (I/O) ports, a column array to serve as frits for the cation exchange resin, and a 50 μL cavity to hold the resin (Fig. S1). Following fabrication, the master was functionalized with a hydrophobic, oleophobic coating of FluoroPel 800 (Cytonix) to facilitate delamination of the PDMS from the mold. Using this master mold, microfluidic channels were prepared by degassing two component PDMS (10:1, Dow SYLGARD) and curing at 65°C overnight. Finally, fluidic access ports were punched in the PDMS, and the final devices were sealed by plasma bonding to a glass slide.

The cation exchange resin was hydrated in DI water for 2 h before manual transfer in a slurry to the chip. For initial experimentation and trend discovery, phenylalanine (phe, 100 mM) was spiked into concentrated salt solutions that may be present on Europa: sodium chloride (NaCl, 5M), calcium chloride (CaCl_2_, 5M), and magnesium sulfate (MgSO_4_, 2.5 M). All stock chemicals were purchased from Millipore Sigma, and stock solutions were prepared in DI water and stored between 0°C and 5°C.

**FIG. 1.**
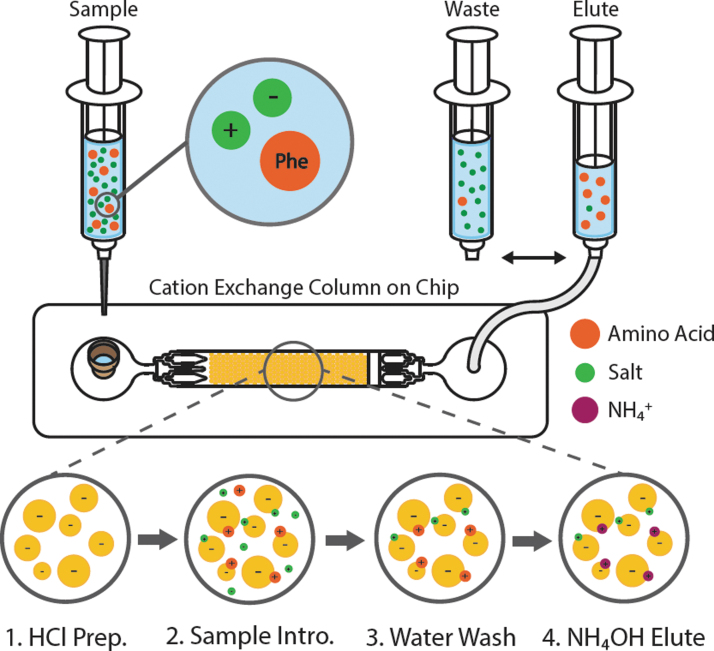
Microfluidic, cation exchange process for desalting a sample containing amino acids. Sequential syringes are introduced as follows: (1) 10 mM HCl to prepare and deprotonate the resin, (2) sample with amino acids that binds to the resin along with higher-affinity cations, (3) DI water to remove excess salts and contaminants, and (4) 1 M NH_4_OH to selectively elute amino acids. A waste syringe collects eluent from steps 1–3, while the elute syringe collects step 4.

To assess performance at high salinity, samples were prepared such that the cation loading was above resin binding capacity (Equation S1 and Table S2), and the ratio of phe to cation ranged from 1:25 to 1:500. Samples were prepared by mixing 50 μL phe with varying volumes of a single salt solution (25–250 μL for a total sample volume of 75–300 μL) and then acidified with 1 N HCl by adding 50 μL per mL of solution, bringing the pH of the sample below 2 (Table S3). For each run, a fresh volume of resin was loaded into the chip bed and flushed with DI water to wet all fluidic lines. During the chromatographic separation, 1 mL sterile luer lock syringes were loaded with buffer and sample and sequentially introduced to the chip as follows: (1) 500–725 μL of 10 mM HCl, (2) 75–300 μL sample (sum of volumes in steps 1 and 2 equaled 800 μL), (3) 800 μL DI water, and (4) 800 μL 1 M NH_4_OH (Fig. 1). At the outlet of the chip, six 400 μL aliquots were collected and analyzed for phe and salt concentrations. Phenylalanine concentration was determined by absorbance using an ultraviolet, visible, and near infrared spectrophotometer (Agilent Technologies, Cary Series) at 258 nm with a 1 mL quartz cuvette. Resulting salt concentration was estimated with a conductivity meter (FiveEasy Benchtop F30 Meter, Mettler Toledo), which measures from 0.01 μS/cm to 200 mS/cm with an accuracy of ±0.5%. The probe was calibrated by using a 1413 μS conductivity standard (Mettler Toledo) before each use.

### 2.3. Cartridge assembly and testing

To improve the durability of the device and its I/O interfaces, the final assembly consisted of a reusable polyetheretherketone (PEEK) based CapTite Cartridge (LabSmith) with 10 μm polyester sample filters (LabSmith) (see details in Discussion section 4.1). This change allowed for consistent resin loading and faster iteration between subsequent runs. With the new cartridge, we performed three different experiments with mixtures of amino acids. The first experiment acted as a salt-free control and introduced 1 mL of 4 μM amino acids in DI water. The second experiment evaluated amino acid competition with excess salt, where “excess” is a concentration that exceeded the resin binding capacity, and introduced 1 mL of 100 μM amino acids in a terrestrial ocean solution. The third experiment determined whether enrichment, defined herein as both amino acid purification and concentration, was possible with both excess amino acids and salts. This final experiment introduced 10 mL of 100 μM amino acids in a terrestrial ocean solution.

Samples for the amino acid mixture experiments were prepared by spiking a 17-amino-acid standard (Millipore Sigma) into DI water and acidifying with 1 N HCl to a pH below 2 with Hydrion test strips. The final concentration of amino acids in the control experiment was 4 μM, except cysteine was 2 μM due to its comparatively low solubility in water from the initial stock solution. Samples for the ocean world analog and the enrichment experiments were prepared by spiking 100 μM amino acids (50 μM cysteine) into a terrestrial ocean sample collected from Cape Henlopen, Delaware, USA. Figure S2 provides details of the sampling location. The ocean sample salinity was analyzed by inductively coupled plasma mass spectroscopy (Caliber Analytical Services/ICP-MS).

Mixtures of amino acids require derivatization and separation before analysis with other methods (*e.g.,* GC-MS, HPLC). We used a Waters Acquity Arc System HPLC coupled to a 2998 photodiode array (PDA) detector. Details on the analytical column, method, and analysis are included in Table S4. Using this method, we improved the sensitivity of amino acid detection by 3 orders of magnitude compared to ultraviolet and visible (UV-vis) detection, that is, to a level of quantitation (LOQ) of 2.5 μM.

To reduce manufacturer contamination (reported as <100 microorganisms per gram) with the increased sensitivity of HPLC detection, the resin was first purified by centrifuging three times at 5000 rpm for 15 min in 1 M NH_4_OH. Supernatant was discarded and replaced with fresh NH_4_OH between each cycle. This decontamination process was optimized through trials of several alternative purification techniques shown in Table S5. After loading into the PEEK cartridge, the whole system was washed with several milliliters of DI water. For all three experiments, a fresh volume of resin was loaded into the chip bed, and we conducted a preliminary blank run with acidified water as the sample for a background baseline of resin contaminants. For each separation analysis, a syringe pump (Model # 78-0100I, Fisher Scientific) set to 0.2 mL/min (∼6.7 mbar pressure, Table S6) introduced (1) 800 μL of 10 mM HCl, (2) 1 mL of sample (10 mL of sample for the enrichment study), (3) 800 μL of DI water, (4) 800 μL of 1 M NH_4_OH followed by a 5 min residence time, (5) 800 μL of 1 M NH_4_OH, and (6) 800 μL of DI water added sequentially to the cartridge.

The outlet was separated into waste (HCl + H_2_O) and elute (NH_4_OH + collected amino acids), and aliquots were dried down in a vacuum oven at 60°C and 85 kPa to remove ammonium hydroxide from solution, which interferes with derivatization, before reconstituting in 800 μL purified water for derivatization and HPLC analysis. An image of the cartridge test assembly is shown in Fig. S3 with a small volume (17 μL) flow-through conductivity probe (ET916, eDAQ) attached to the outlet to identify bubbles and confirm salt reduction before beginning elution. A comparison of drying methods and their impact on phe retention is shown in Table S7. The bead bed capacity was not overloaded with excess salts for the amino acid mixture experiments, but it was overloaded by more than four times in the terrestrial-ocean experiments (Table S8). This ensured that amino acids would be above HPLC LOQ, since the increased salt competition in the terrestrial ocean sample reduces the resin recovery efficiency of some amino acids.

## 3. Results

### 3.1. Chip + UV-vis experiments

We used initial desalination experiments to investigate the effect of salt type and ionic concentration on phenylalanine recovery with a 50 μL microfluidic cation exchange chip. Salt solutions of concentrated NaCl, MgSO_4_, and CaCl_2_ allowed for assessment of single cation binding affinity at increasing concentrations and its effect on separation. Volumes introduced overloaded the bead bed with excess salt to simulate the expected high-salinity environment of ocean worlds. To ensure phenylalanine was fully protonated, the sample was buffered to a pH of 2, which is well below the acid dissociation constant (pKa) of the carboxyl group. By implementing tight controls on sample preparation, separation, and analysis during the first experiment, the dominant variable monitored was sample composition.

**FIG. 2.**
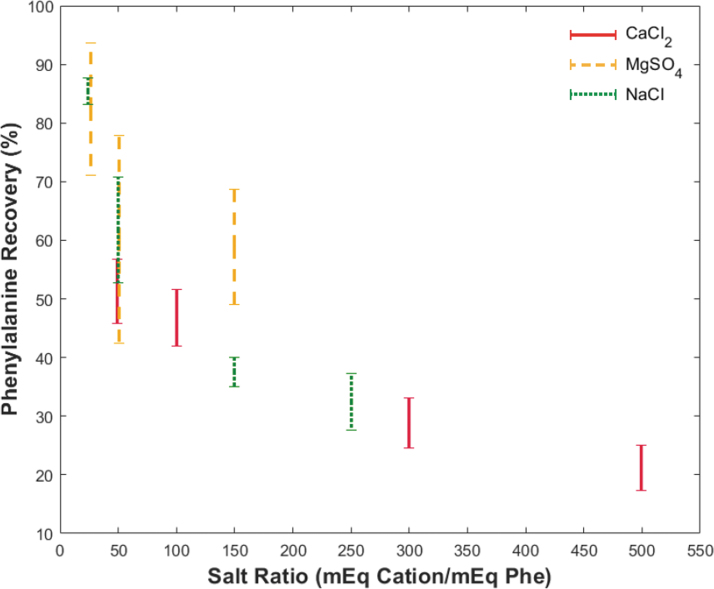
Decreasing phenylalanine recovery with increasing salt ratio. The vertical axis shows phenylalanine recovery as a percentage of amino acid introduced, and the horizontal axis shows the ratio between milliequivalents of cation introduced (Ca^2+^, Mg^2+^, and Na^+^) and phenylalanine introduced. Vertical bars represent eluted phenylalanine recovery range from a single experiment based on the 400 μL collection volume. The bottom marker indicates the full introduction of NH_4_OH elution buffer to the chip, and the top marker indicates a subsequent DI water flush to remove remaining elution buffer from the chip.

Figure 2 shows the effect of cation type and concentration on phe recovery. The horizontal axis shows increasing salt ratio defined as the milliequivalents (mEq) of cation divided by the milliequivalents of phe. All tests utilized an overloaded bead bed (Table S2) to evaluate separation limits, where the mEq of charged species outnumbered the binding sites available (0.085 mEq). Phenylalanine recovery percentage allows for comparison of the abundance of phe in the eluent to that of the calculated phe introduced. Each vertical bar represents a single salt-free recovery range that arises from the large aliquot sizes needed for UV-vis detection (400 μL) and the fluidic dead volume of the chip and its I/O connections (∼300 uL). Phenylalanine is determined “salt-free” when the eluent conductivity dropped below 1.8 mS/cm, estimated from the conductivity of 1 M NH_4_OH elution buffer and charged amino acids in solution. The actual salt-free recovery value lies between these bounds, as the chip dead volume is less than the collected aliquot volume. Table 1 details values for phe recovery and reduced elution conductivity that correspond to the vertical bar markers in Fig. 2 based on salt type and volume introduced. Initial conductivities of all samples introduced exceeded the conductivity probe detection range (>200 mS/cm) due to the presence of HCl.

**Table 1.** Impact of Cation Affinity on Separation Using the Microfluidic Chip

Salt-solution volume (μL)	Cation [Affinity]					
	5 M Na^+^ [1.5]		2.5 M Mg^2+^ [2.5]		5 M Ca^2+^ [3.9]	
	Eluted phe (%)	Conductivity (mS/cm)	Eluted phe (%)	Conductivity (mS/cm)	Eluted phe (%)	Conductivity (mS/cm)
25	83–88	1.3–1.8	71–94	0.6–0.9	46–57	0.1–0.3
50	53–71	0.4–1.3	42–78	0.6–0.8	42–52	0.6–2.3
150	35–40	1.8–2.8	49–69	0.6–0.9	25–33	0.9–2.9
250	28–37	1.7–2.3	—	—	17–25	1.5–7.0

The “Eluted phe” column shows the range of the original phenylalanine (phe) recovered, and the “Conductivity” column reflects the conductivity range of salts remaining in the purified sample.

Recovery of phe with Na^+^ ranged from 28% with a high salt loading to 88% with a lower salt loading. In all experiments, the elution conductivity was below 2.8 mS/cm, indicating at least a 70 × reduction from the initial sample introduced. For Mg^2+^, normalized recoveries spanned 42–91%, with a maximum conductivity of 0.9 mS/cm (∼200 × reduction). Due to the lower solubility limits of MgSO_4_, the 250 μL separation was not attempted. Lastly, the highest affinity cation, Ca^2+^, had the lowest phe recovery ranges from 17–57% with a max conductivity of 7 mS/cm (∼30 × reduction) at the highest salt loading. Table S9 provides a rough estimate of eluent salt concentrations based on molar conductivity calculations of pure salt solutions.

### 3.2. Cartridge + HPLC-PDA experiments

**FIG. 3.**
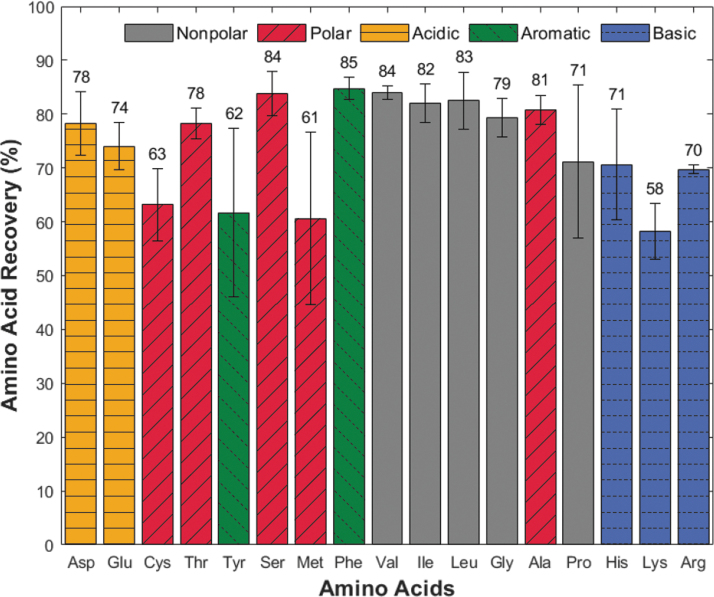
Average recovery of 17 amino acids without salts in the 4 μM control experiment is independent of isoelectric point (pI) or amino acid side chain chemistry (R group). The horizontal axis plots amino acids by increasing pI, and colored bars identify amino acid R group. Values above the bars identify the mean value of triplicate runs, and vertical error bars represent one standard deviation in the positive and negative directions. Ala = alanine; Arg = arginine; Asp = aspartic acid; Cys = cysteine; Glu = glutamine; Gly = glycine; His = histidine; Ile = isoleucine; Leu = leucine; Lys = lysine; Met = methionine; Phe = phenylalanine; Pro = proline; Ser = serine; Thr = threonine; Tyr = tyrosine; Val = valine.

To evaluate amino acid competition with the resin, all remaining analyses utilized a mixture of 17 amino acids spiked into either DI water or a terrestrial ocean sample that acted as an ocean world analog. The latter contained 2000 ppm Ca^+^, 510 ppm Mg^2+^, 7300 ppm Na^+^ evaluated by ICP-MS (Caliber Analytical Services), and possible other dilute minerals, organic compounds, or biological material found in Earth's ocean (Biller *et al.,* 2018). For these tests, we transitioned to a 50 μL PEEK cartridge (described in Section 2.3), which allowed us to load the resin slurry more easily to a consistent bed volume, and HPLC-PDA analysis to identify mixtures of amino acids with increased sensitivity (LOQ ∼2.5 μM). With the improved repeatability of the cartridge loading and the improved sensitivity of the HPLC-PDA analysis, we conducted three experiments to characterize the recovery of amino acids on the resin with challenging sample compositions. Here, the binding kinetics of these systems were much more complex, as each amino acid must compete with one another and other charged species in the ocean sample for binding sites. The first experiment consisted of a standard mixture of 17 common amino acids spiked into DI water at 4 μM. This was a control sample near the LOQ for HPLC-PDA, to evaluate amino acid competition without confounding salts (Fig. 3).

The 4 μM amino acid mixture experiment had average individual amino acid recoveries ranging from 58% (lys) to 85% (phe), with an average recovery of 74% across amino acids. The average standard deviation in triplicate runs for each amino acid is 6% with no clear trend across isoelectric point (pI) or side chain (R group, indicated by colored bars). Amino acids with standard deviations above 10% include proline (14%), methionine (16%), and tyrosine (16%), which may be due to resin contaminant peaks (see Discussion section 4.3).

**FIG. 4.**
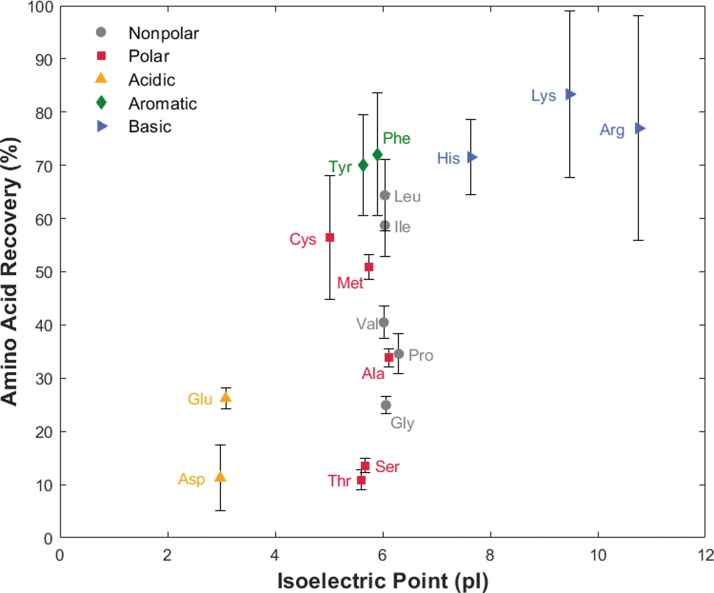
Average recovery of 17 amino acids in an ocean world analog sample is dependent on amino acid side chain chemistry (colored markers) rather than isoelectric point (horizontal axis order). With increased competition for binding sites, recovery percentages vary more widely between amino acids and sequential experiments. Vertical error bars represent one standard deviation in the positive and negative direction from the triplicate study. Abbreviations are the same as in Fig. 3.

The 100 μM ocean world analog experiment demonstrates the competition between amino acid and salt mixtures by spiking the same 17-amino-acid standard solution into a terrestrial ocean sample (collected from Cape Henlopen, DE). The higher concentration of amino acids ensured amino acid detectability with competing cations. Figure 4 shows the amino acid recovery based on both increasing pI and R group chemistry. The average individual recoveries ranged from 11% (asp, thr) to 83% (lys), with an average recovery across all amino acids of 47%. The average standard deviation between triplicate runs for each amino acid was 7%, which is similar to that of the control experiment. Amino acids with standard deviations above 10% include cysteine (11%), phenylalanine (11%), lysine (15%), and arginine (21%), which were different from the amino acids in the control experiment, indicating experimental error rather than poor performance of select amino acids (see Discussion section 4.3).

**FIG. 5.**
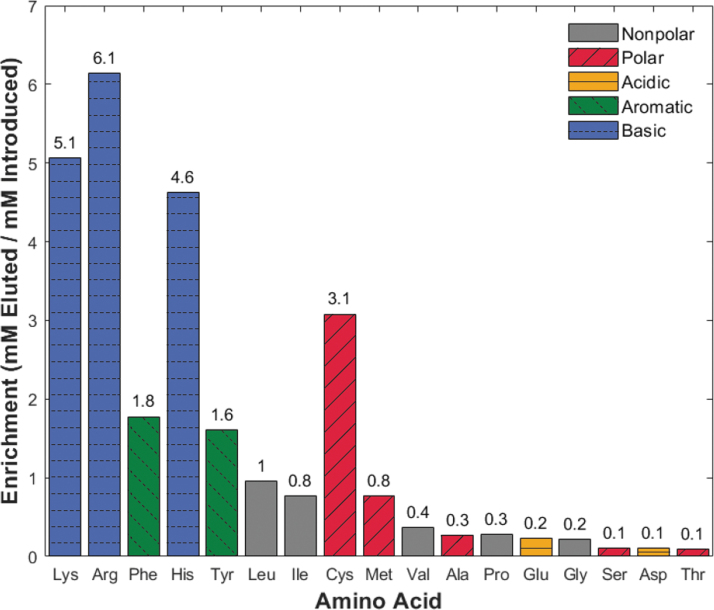
Enrichment (mM recovered/mM introduced) of 17 amino acids in an ocean world analog sample is possible for several charged amino acids. The horizontal axis arranges amino acids by decreasing recovery percentage from Fig. 4 to demonstrate the impact of increased amino acid competition. Abbreviations are the same as in Fig. 3.

Additionally, we performed a third experiment to determine whether enrichment, that is, purification and concentration of amino acids, could take place with excess cationic species (salts and amino acids) competing for binding sites. Separation utilized 10 mL of terrestrial ocean sample spiked with 100 μM of the 17-amino-acid mixture. The amino acids were eluted in 800 μL of NH_4_OH, introducing a theoretical 12.5 × sample concentration. Figure 5 shows the enrichment of each amino acid by dividing the final eluted concentration by the initial concentration for each amino acid. The horizontal axis lists amino acids in order of decreasing amino acid recovery from Fig. 4. This trend illustrates the differences between amino acid competition with an excess of salts (Fig. 4) versus amino acid competition with an excess of salts and amino acids (Fig. 5). The enrichment experiment results show amino acid losses from 0.1 × (ser, asp, thr) to concentrations up to 6.1 × (arg). Seven of the 17 amino acids demonstrated enrichment (mM recovered/mM introduced ≥1), indicating higher selectivity for the resin.

## 4. Discussion

### 4.1. Microfluidic design and analysis technique

Initial chip experiments leveraged PDMS and glass chips useful for rapid prototyping, and the transparent materials allowed for bubble detection and avoidance upon resin loading. However, PDMS will readily absorb small molecules, possibly even including biological material (Toepke and Beebe, 2006). Additionally, PDMS is not a space-qualified material and has limited reusability due to poor mechanical robustness. Therefore, in later experiments, we transitioned to a commercial microfluidic cartridge made of PEEK for its small form factor and threaded I/O interfaces. This change also allowed for faster iteration between experiments and consistent resin loading between runs.

Additionally, changes to the analysis technique were required to evaluate complex mixtures of amino acids. Preliminary experiments utilized phenylalanine with UV-vis analysis because derivatization was not required with the aromatic side chain of phe. However, the UV-vis detection limit of LOQ (∼1 mM) is many orders of magnitude higher than what is needed (1 nM) for analog space samples (Hand *et al.,* 2017), and phe is not one of the biogenic indicator amino acids of interest (Creamer *et al.,* 2017). Therefore, we transitioned to the more-sensitive HPLC-PDA analytical technique, which can also identify the type and concentration of each amino acid. The increased detector sensitivity also required rigorous experimental controls, including biosafety hoods, analytical-grade reagents, bead purification procedures, and control experiments between triplicate runs.

### 4.2. Cation competition

The first experiment (Fig. 2) utilized simplified binding kinetics by investigating a single high-yield amino acid (phe) and its competition with an excess of a single cation (Na^+^, Mg^2+^, or Ca^2+^). Each cation tested has a different relative selectivity for the resin (1.5, 2.5, and 3.9 for Na^+^, Mg^2+^, and Ca^2+^, respectively), which is defined as the equilibrium ratio of ions bound on the resin to free ions in solution normalized by the native hydrogen (H^+^) form. During cation exchange, higher-selectivity ions exchange with bound ions at the same concentration or with higher concentrations of lower-selectivity ions. The latter explains why introducing NH_4_OH at low concentrations does not elute all higher-selectivity ions (Mg^2+^, Ca^2+^) from the resin. The former explains how the NH_4_^+^ ion (relative selectivity of 1.95) in the elution buffer replaces deprotonated amino acids at high pH. When evaluating Fig. 2, it is important to note that all resin beds were overloaded (Table S2) to test the limits of separation. Even so, these preliminary experiments confirmed that 17% phe recovery in the presence of excess salt and in a continuous-flow device was possible, even when high-affinity Ca^2+^ was in excess of 500:1. This experiment highlights the importance of choosing appropriate resin volumes to accommodate the worst-case ocean world environments for the chosen sample volume.

### 4.3. Amino acid competition

The control experiment (Fig. 3) characterized relative recovery among different types of amino acid with an excess of resin binding sites (0.085 mEq at equilibrium). When looking at the amino acid recoveries, they are consistent, ranging from 58% to 85% without any statistically significant trend (*P =* 0.373) based on pI or R group (Fig. S4). These values are below those established in literature with the same resin (∼90%, Takano *et al.,* 2010), but this is to be expected for a small-scale continuous flow-through system that is not in equilibrium (Wang *et al.,* 1989; Melis *et al.,* 1996). The standard deviation of the triplicate runs for each amino acid averaged 6%, indicating good control over input variables between experiments. The higher variability of the methionine, proline, and tyrosine data is likely due to background interference peaks coming off the resin, as seen in the blank runs (Fig. S5). Final concentrations subtracted background contamination seen in blank samples run on the same bead bed prior to each experiment.

To evaluate the influence of amino acid competition in a complex salty sample, we performed the 100 μM ocean world analog experiment (Fig. 4). To ensure eluted amino acids were above the HPLC-PDA limit of quantitation (LOQ) with increased competition for binding sites, we increased the sample's amino acid concentration by a factor of 25 based on recoveries in both the control (Fig. 3) and salt overloading experiments (Fig. 2). Unlike the control experiment, Fig. 4 shows clear binding preferences for amino acids based on their side chain (*P =* 0.005, Fig. S6). Additionally, the 7% average error in this experiment was slightly larger than the control, with four different amino acids having standard deviations greater than 10%. We attributed this to small variations in the introduced sample volume between trials rather than background contamination, based on the lower recovery percentages of high-affinity amino acids conversely compared to the higher recovery percentages of low-affinity amino acids in elute 3 when contrasted to elutes 1 and 2 (Fig. S7). Ongoing work to automate the sample and buffer fluid introduction will address this issue in future design iterations.

In Fig. 4, the amino acids are grouped into five categories for this study: (1) nonpolar (gray circle), (2) polar (red square), (3) acidic (yellow up-facing triangle), (4) aromatic (green diamond), and (5) basic (blue right-facing triangle). When plotted against pI, amino acids with a positively charged amine in their side chain (group 5) had higher recoveries than amino acids with negatively charged carboxylic acid in their side chain (group 3). This is due to electrostatic repulsion between acidic amino acids and the stationary phase, causing some to elute prior to reaching the resin void volume (Alpert, 2008). Additionally, π-π stacking interactions between aromatic side chains (group 4) with the backbone of the resin (polystyrene divinylbenzene) lead to high recoveries. Similarly, the number of carbons on nonpolar amino acid side chains (group 1) increases with recovery. In both cases, a favorable interaction of the hydrophobic side chain with the hydrophobic backbone of the resin causes increased binding affinity (Dye *et al.,* 1990; Cheng *et al.,* 2006). We postulate that the polar groups have lower recoveries because the side chain groups interfere electrostatically with sulfonic acid and prefer the mobile aqueous phase. Of the polar amino acids, cysteine and methionine exhibited the highest binding affinity, attributed to the electrostatic interaction of their thiol and sulfonate side chains respectively with the resin's sulfonic acid functional group.

As a final experiment, we sought to enrich amino acids from an ocean world analog sample with the presence of both competing cations and amino acids with an overloaded bead bed. We tested this by loading a larger volume (10 mL) onto the column and eluting in a smaller volume (800 μL), corresponding to a possible 12.5 × amino acid concentration. This capability is particularly important when biomass concentrations are below the LOQ for downstream analytical instruments. Figure 5 indicates that all the high-recovery (>50%) amino acids from Fig. 4 (lys–met) have final concentrations close to, or greater than, their introduced concentrations (0.8–6.1 × ). Notably, the basic and aromatic amino acids (lys, arg, phe, his, and tyr) show enrichment between 1.6 × and 6.1 × . The low mM recovered/mM introduced values (0.1–0.4 × ) of low-recovery (<50%) amino acids in Fig. 4 (val–thr) provide additional evidence that they are being outcompeted for binding sites. In fact, we found that the higher-affinity amino acids are statistically more likely to enrich than their low-affinity counterparts in a terrestrial ocean solution (*P* = 0.003, Fig. S8). These results demonstrate that targeted concentration is possible for some high-affinity amino acids that outcompete both salts and other amino acids for binding sites. Especially promising is that arginine, lysine, histidine, and cysteine all enrich more than three times their initial concentration in the sample. These amino acids have a high relative abundance in biotic samples on Earth due to their role in catalyzing reactions in active enzymatic sites (Georgiou, 2018; Glavin *et al.,* 2020). These three amino acid competition experiments lay the foundation for increasingly sophisticated studies by demonstrating the importance of understanding cation exchange kinetics with de-salting resins. We show that amino acid retention depends on both total amount in the sample and relative concentration to other cationic species. Future proposals applying this technique must ensure that either resin bed sizes are sufficiently large to account for cation competition (see Discussion section 4.2) and amino acids of differing binding affinities (Table S10) or re-introduce the initial cation-reduced sample and waste streams to subsequent column(s). Such considerations are essential for downstream analyses that rely on amino acid abundance ratios to differentiate between biotic and abiotic origins.

## 5. Conclusions

The future of *in situ* amino acid detection will require purification of diverse and often *a priori* unknown, icy samples to achieve mission sensitivity and specificity targets. This study demonstrates a novel modular cation exchange separation system for ocean world environments capable of pairing with multiple downstream analyses. Experiments with a model amino acid (phenylalanine) and salts determined recovery percentages based on milliequivalents of cations introduced (Na^+^, Ca^2+^, and Mg^2+^) and validated the efficacy of this approach at high cation competition ratios (up to 500:1). Additionally, this work investigated competitive binding in mixtures of 17 amino acids and illustrated the importance of side chain chemistry on recovery ratios in a complex terrestrial ocean field sample. The trend persisted in the enrichment study, where only amino acids with recovery ratios greater than 50% in the ocean world analog experiments (Fig. 4) demonstrated similar (0.8 × ) or enhanced (up to 6.1 × ) amino acid binding (Fig. 5). This amino acid desalination device demonstrates a simple, small form factor, and sample agnostic method to improve the limit of detection for amino acids in ocean worlds. Current work focuses on improving automation and spaceflight-readiness and implementing design modifications for integration with heritage downstream analyses such as gas chromatography–mass spectrometry (Blase *et al.,* 2020a, 2020b), capillary electrophoresis, and others in development.

## Supplementary Material

Supplemental data

## Supplementary Material

Supplementary Equation S1

## Abbreviations Used

DI: deionized
GC: gas chromatography
HPLC: high-performance liquid chromatography
ICP-MS: inductively coupled plasma mass spectroscopy
I/O: inlet/outlet
LOQ: level of quantitation, limit of quantitation
MOMA: Mars Organic Molecule Analyser
MS: mass spectrometry
PDA: photodiode array
PDMS: polydimethylsiloxane
PEEK: polyetheretherketone
phe: phenylalanine
pI: isoelectric point
SAM: Sample Analysis at Mars
UV-vis: ultraviolet and visible

## Notes

Associate Editor: Don Cowan
