# Supplementary material for: Microfluidic Chromatography for Enhanced Amino Acid Detection at Ocean Worlds
Source: Astrobiology. 2022 Sep 5;22(9):1116–28. doi: 10.1089/ast.2021.0182 (PMC9508454; doi:10.1089/ast.2021.0182)
Supplement: Supplemental data [file Supp_Data.pdf]

Supplemental Information

Microfluidic Chromatography for Enhanced Amino Acid Detection at Ocean Worlds

Section 1: BioRad Resin

Table S1. Previous work utilizing BioRad AG50 resin for space-relevant amino acid purification

Paper	Sample(s)	BioRad Resin	Elution Buffer	Derivatization	Analysis Technique
(Zhao and Bada, 1995)	Mixtures of α-dialkylamino acids	AG50-X8	HCl	OPA/NAC	HPLC-FD
(McDonald and Bada, 1995)	Martian meteorite EETA 79001	AG50-X8	NH_4OH	OPA/NAC	HPLC-FD
(Bada, 1998)	Martian meteorite ALH84001	AG50-X8	Not known	OPA/NAC	HPLC-FD
(Pizzarello and Cronin, 2000)	Murchison and Murray carbonaceous chondrites	AG50-X4	NH_4OH	TAI/PPI	GC/MS
(Takano et al., 2003)	Deep subterranean hydrothermal vent in Toyoha mine (Hokkaido, Japan)	AG50-X8	NH_3	OPA/NAC	HPLC-FD
(Pizzarello et al., 2004)	Murchison meteorite	AG50-X4	NH_4OH	TFAA/IPA	GC-MS, GC-C-IRMS
(Skelley et al., 2005)	Soil samples (Atacama desert, Chile) and Jarosite (Panoche Valley, United States)	AG50-X4	NH_4OH	OPA/NAC	HPLC, Mars Organic Analyzer (MOA)
(Glavin et al., 2006)	Antarctic CM2 chondrites ALH83100 and LEW90500	AG50-X8	NH_4OH	OPA/NAC	HPLC-FD, LC-ToF-MS
(Botta et al., 2007)	Antarctic CM1 chondrites MET01070, ALH88045, and LAP02277	AG50-X8	NH_4OH	OPA/NAC, PFP/IPA	HPLC-FD, GC-ToF-MS
(Martins et al., 2007)	Antarctic CR meteorites EET92042, GRA95229, and GRO95577	AG50-X8	NH_4OH	OPA/NAC, TFAA/IPA	HPLC-FD, GC-MS, GC-C-IRMS
(Amashukeli et al., 2007)	Soil samples from the Chilean Atacama Desert	AG50-X8	NH_4OH	AccQ·Tag™ method	HPLC
(Liu et al., 2008)	Mars analogue samples from Mono Lake (CA), evaporate lake bed (Death Valley, CA), Haughton	AG50-X8	NH_4OH	NFPA/ACN	HPLC-ESI-MS/MS

	Crater (Devon Island, Canada)				
(Glavin et al., 2010)	Nine CI, CM, and CR carbonaceous chondrites including Orgueil, MET01070, SCO06043, GRO95577, Murchison, LON94102, LEW90500, EET92042, and QUE99177	AG50-X8	NH_4OH	OPA/NAC	UPLC-FD/ ToF-MS
(Takano et al., 2010)	Calcareous, siliceous, rock and sediment samples, aggregated microbial samples, and biological soft tissue samples	AG50-X8	NH_3	Not known	GC/FID and GC/MS, GC/C/IRMS
(Martins et al., 2013)	Icy mixtures subjected to hypervelocity impact shock waves	Did not specify	NH_4OH	TFAA/IPA	GC-MS
(Elsila et al., 2016)	Seven Apollo 15, 16, 17 lunar regolith samples	AG50-X8	NH_4OH	OPA/NAC, TFAA/IPA	LC-FD/ToF-MS, GC-MS/IRMS

Section 2: Chip Tests

Figure S1. Image of cation exchange resin chip

The initial PDMS chip design included an (a) inlet port, (b) 50 µL bead bed cavity, (c) micro-column frit array, (d) outlet port, and (e) branching regions. The micro-column frit array held the bead bed in place while the branching regions ensured an even flow distribution across its relatively wide channel. The final footprint measured approximately 7 x 1 cm with a 150 µm channel depth.

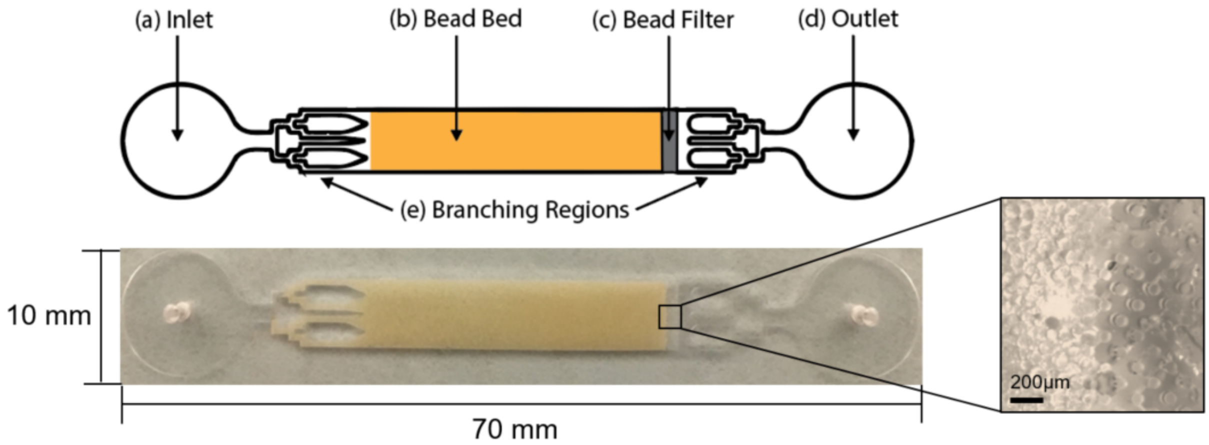

Equation S1. Cation capacity calculations for a 50μL bead bed

$$\textit{bead bed capacity} - \text{ mEq phenylalanine} = \text{mEq remaining}$$

$$\left(50\ \mu\text{L} * \frac{1\ \text{mL}}{1000\ \mu\text{L}} * \frac{1.7\ \text{mEq}}{1\ \text{mL}}\right) - \left(50\ \mu\text{L} * \frac{1\ \text{L}}{10^6\ \mu\text{L}} * \frac{100\ \text{mmol}}{1\ \text{L}} * 1[\text{valence}]\right) = 0.08\ \text{mEq}$$

This paper calculated the theoretical binding capacity for 50 µL of resin to be 0.085 mEq from the reported BioRad AG50W resin equilibrium cation binding capacity (1.7 mEq/mL). All chip tests introduced 50 µL of 100 mM phenylalanine (0.005 mEq), reserving only 0.080 mEq for salts.

Table S2. Calculation of salt overloading in UV-vis chip experiments

During the UV-vis chip experiments, overloading calculations divided the milliequivalents of salts introduced by the milliequivalents of binding sites remaining (0.08 mEq). Values greater than 1.0 indicate overloaded bead beds.

**Cation**	**5M Na^+**		**2.5 M Mg^{2+}**		**5 M Ca^{2+}**	
Volume (µL)	mEq salt	mEq salt/mEq remaining	mEq salt	mEq salt/mEq remaining	mEq salt	mEq salt/mEq remaining
25	0.0125	0.16	0.025	0.31	0.025	0.31
50	0.025	0.31	0.05	0.63	0.05	0.63
150	0.075	0.94	0.15	1.88	0.15	1.88
250	0.125	1.56	N/A	N/A	0.25	3.13

Table S3. Dependence of pH on separation

Tests were performed with a 50:1 $MgSO_4$ to phenylalanine sample where the pH was buffered to 2, 4, and 6 with HCl or NH_4OH. These results indicate similar separation efficiencies occur when the sample pH is below the isoelectric point for phenylalanine (pI ~ 5.5). Above this value, the phenylalanine yield decreases and eluted conductivity increases.

**Sample pH**	**Phe Yield (%)**	**Conductivity (mS/cm)**
pH 2	66 - 94	0.2 – 0.9
pH 4	59 - 95	0.2 – 0.9
pH 6	53 - 76	0.2 – 1.1

Section 3: Cartridge Tests

Figure S2. Ocean analog sample

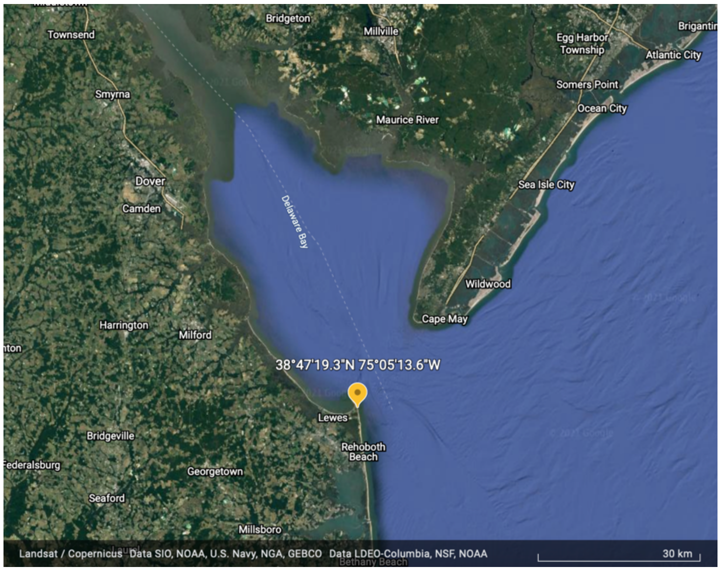

Samples were collected off Cape Henlopen, DE on the ocean side, September 27, 2020 into 50 mL conical tubes (Lat:38.7887, Lon:-75.0871). Samples here are not of as high salinity as average seawater due to the proximity to the Delaware River outlet.

Table S4. HPLC Analysis Parameters

We performed HPLC analysis using a Waters Acquity Arc system coupled to a 2998 Photodiode Array (PDA) detector (HPLC-PDA). The HPLC analysis utilized gradient elution, with mobile phases consisting of an acetate-phosphate buffer solution, pH 5.03 (MPA) and a mixture of 60% Acetonitrile and 40% Water (MPB). The acetate-phosphate buffer was prepared by appropriately diluting the AccQ-Tag Eluent A concentrate with water and adjusting the pH with phosphoric acid or sodium hydroxide, as needed, to ensure that the pH was within the target value. Retention time and peak area were determined by Empower 3 Chromatography Data Software and amino acid concentration was obtained from a calibration curve constructed by plotting the peak area versus concentration for each amino acid.

**Parameter**	**Setting**
Analytical Column	4 µm C18, 3.9 x 150 mm
Column Temperature	37°C
Autosampler Temperature	20°C
Detection	248 nm
Flow Rate	1.0 mL/min
Injection Volume	10 µL

Table S5. BioRad resin purification trials

To reduce BioRad resin contamination as determined by HPLC-PDA analysis, permutations of 1, 3, 4, and 5 washes in 1 M HCl, 1 M NH_4OH and NERL reagent grade water were performed for periods ranging from 15 minutes to 1 hour. During each step, the wash decant was analyzed against a seventeen amino acid standard. All washing steps, excepting those using HCl, drop the concentration of residual amino acids

below the 2.5 µM quantitation limit. Test results show that three fifteen minute NH_4OH washes (Trial 10) provide the best contamination removal. Though Trial 8 initially seems like an equal performer to Trial 10, subsequent washes (shown in Trial 9) indicate that amino acids remained bound. The denotation NQ, or not quantitated, refers to contaminants that were present at levels too low to be reliably integrated by the HPLC. Additionally, each sample contained non-quantifiable levels of both NH_3, an evaporation byproduct, and AMQ, the derivatization reagent.

Trial #	Wash Time	Wash Solution	Amino Acids (µM)	
1	1 hour	NERL Water	Tyrosine	0.754
			Alanine	0.420
			Threonine	NQ
			Serine	NQ
			Glycine	NQ
			Proline	NQ
2	1 hour	1M NH_4OH	Alanine	0.337
			Glycine	0.033
			Phenylalanine	0.012
			Tyrosine	NQ
			Serine	NQ
			Proline	NQ
3	1 hour	NERL Water	Threonine	1.590
	1 hour	NERL Water	Glycine	0.079
	1 hour	NERL Water	Tyrosine	NQ
			Serine	NQ
			Alanine	NQ
			Proline	NQ
4	1 hour	1M NH_4OH	Threonine	1.604
	1 hour	NERL Water	Tyrosine	0.198
	1 hour	NERL Water	Serine	NQ
			Glycine	NQ
5	1 hour	1M NH_4OH	Threonine	0.195
	1 hour	1M NH_4OH	Tyrosine	NQ
	1 hour	NERL Water	Serine	NQ
			Glycine	NQ
6	1 hour	1M HCl	Threonine	3.440
	1 hour	NERL Water	Histidine	0.704
	1 hour	NERL Water	Tyrosine	0.281
			Glycine	0.051
7	1 hour	1M HCl	Threonine	2.886
	1 hour	1M HCl	Tyrosine	0.358
	1 hour	NERL Water	Glycine	0.130
			Serine	NQ
8	1 hour	1M NH_4OH	Glycine	NQ
	1 hour	NERL Water		
	1 hour	1M HCl		
9	15 mins	1M NH_4OH	Threonine	1.271
	15 mins	NERL Water	Phenylalanine	0.693
	15 mins	1M HCl	Proline	0.612
	15 mins	NERL Water	Glycine	0.324

	15 mins	1M NH_4OH	Glutamine<br>Tyrosine	0.319<br>0.251
10	15 mins	1M NH_4OH	Glycine	NQ
	15 mins	1M NH_4OH		
	15 mins	1M NH_4OH		

Figure S3. Cartridge test assembly

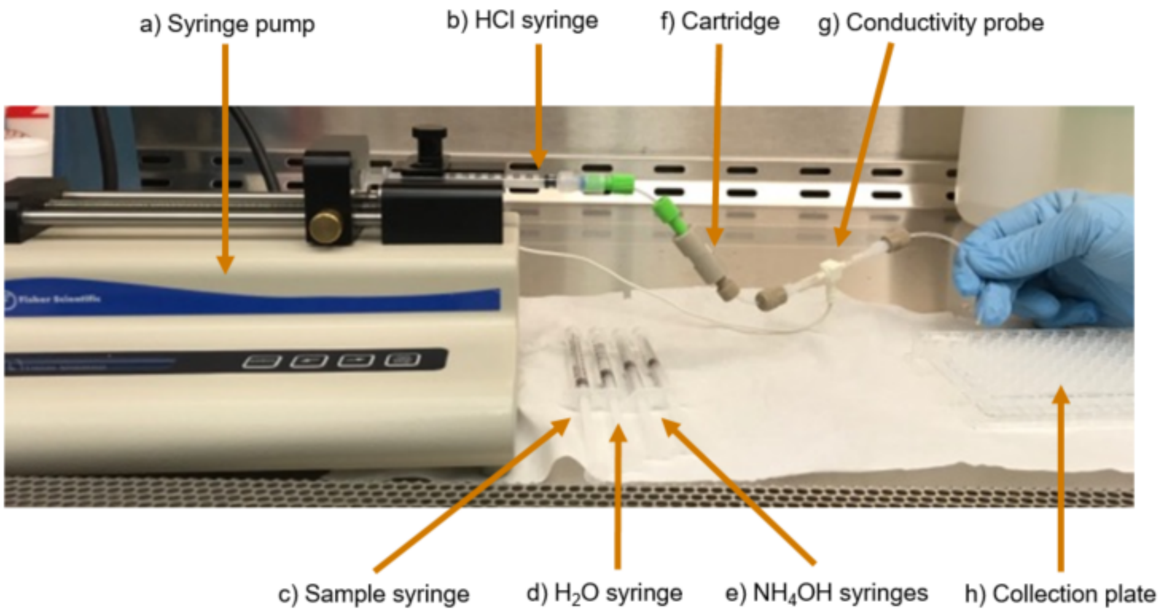

A syringe pump (a) drives the separation by sequentially introducing sample and buffer syringes (b-e). The cartridge (f) contains cation exchange resin, and a downstream conductivity probe (g) records the relative salinity. A 96 well plate (h) collects samples before subsequent HPLC-PDA analysis.

Table S6. Approximating introduction pressure with the Ergun Equation

Although pressure measurements were not performed during separation experiments, we can approximate the pressure drop through the 50 µL cartridge using the Ergun Equation (Worstell, 2016) for the known volumetric flow rate of 0.2 mL/min and other known parameters listed in Table S6. The bead packing density was estimated to be 55% (45% void space), and the average particle size was approximated as 100 µm (63 – 150 µm range as stated for AG50W-X8, Bio-Rad Laboratories). The fluidic resistance scales linearly with column length.

$$\frac{\Delta P}{L} = \frac{150\mu u_0(1-\epsilon)^2}{\varepsilon^3 d_p^2} + \frac{1.75\rho u_0^2(1-\varepsilon)}{\epsilon^3 d_p}$$

Parameter	Value
Volumetric Flow Rate (Q, m^3/s)	3.33E-09
Cartridge Diameter (d, m)	2.05E-03
Cross Sectional Area (A, m^2)	3.30E-06

Height of Bed (L, m)	1.50E-02
Fluid Viscosity (μ, Pa·s)	1.00E-03
Void Space (ε)	0.45
Fluid Superficial Velocity ($u_{0,}$ m/s)	1.00E-03
Particle Diameter (d_p, m)	1.00E-04
Fluid Density (ρ, kg/m^3)	9.97E+02
Pressure Drop (ΔP, Pa)	-667.6

Table S7. Phenylalanine yields with multiple NH_4OH drying procedures

Drying was necessary to remove excess NH_4OH from solution before subsequent derivatization for HPLC-PDA analysis. Drying procedures attempted included heated vacuum evaporation with and without the presence of a high capacity desiccant (Syloid 244FP Silica, W.R. Grace) and positive air displacement on a heated block. The test used 0.3 – 1 mL of 12.5 mM phenylalanine stock solution into three different 4 mL vials, dried them under the conditions mentioned, and rehydrated in the same volume of DI water. The smaller volume of phenylalanine for the heated desiccant trial was due to the long dry times required. Results compared the UV-vis absorbance in the rehydrated samples to the initial stock solution. The method selected was vacuum evaporation without desiccant while heating due to high yield and short dry times. Since the environments for missions that would use this instrument are vacuums, we would use the same drying method for flight, with a vent path providing the needed vacuum.

Drying Technique	Drying Conditions	Initial Sample Volume	Approx. Dry Time	Phenylalanine Yield
Vacuum Oven	25 inHg vacuum and 80°C heat	1 mL	3 hours	87%
Heated Desiccant	60°C heat, and 10 g of Syloid 244FP Silica	0.3 mL	21 hours	93%
Air Dryer	10 – 20 psi of house air, and 60°C heat	1 mL	40 minutes	86%

Table S8. Calculation of overloading in the ocean analog experiments

External ICP-MS analysis of the ocean sample confirmed the presence of Ca^{2+}, Na^+, and Mg^{2+} ions. Based on these values, the calculated 0.37 mEq present in 1mL of solution is approximately four times the theoretical capacity of the resin (0.085 mEq) before spiking with amino acids.

Ocean Analog Sample	mL	mg/mL	g/mol	mmol	mEq
100uM Amino Acid	1	-	-	0.0017	0.002
Ca^{2+}	1	0.2	40	0.005	0.01
Na^+	1	7.3	23	0.32	0.32
Mg^{2+}	1	0.51	24	0.02	0.04
Sum of cations					**0.37**

Table S9. Estimating remaining salt concentrations

For the UV-vis chip experiments, aliquots were tested for conductivity in the eluent (1M NH_4OH, pH ~13) and with varying concentrations of phenylalanine. To approximate the remaining salt concentrations from these measurements, and because all three salts (NaCl, $MgSO_4$, and $CaCl_2$) dissociate strongly in water, we used molar conductivity calculations (Coury, 1999). Here the ionic concentration (C_i), depends on the conductivity (κ), Faraday's constant (F), ion charge (z_i), elementary charge constant (e), solution viscosity (η), and hydrated radius (R_i) for each ion in solution. This estimate assumes cations and anions are present at their correct molar ratios in water in the eluent. To assess the validity of this method, we compared the NaCl conductivities with a standard curve of NaCl in water [Conductivity (mS/cm) = 446.42 x NaCl concentration (M), R^2 = 0.9997 to 0.05M]. The calculated concentrations using the molar conductivity method differed between 16 ± 8 mM for the lower value, and 25 ± 8 mM for the upper value.

$$\kappa = F \sum_i |z_i| \left(\frac{|z_i| e}{6\pi\eta R_i} \right) C_i$$

Volume (µL)	Estimated Salt Remaining NaCl (mM)	Estimated Salt Remaining $MgSO_4$ (mM)	Estimated Salt Remaining $CaCl_2$ (mM)
25	25 - 34	3 - 5	1 - 2
50	8 - 25	3 - 4	5 - 18
150	34 - 53	3 - 5	7 - 23
250	32 - 43	-	12 - 56

Figure S4. ANOVA analysis of 4 µM control experiment

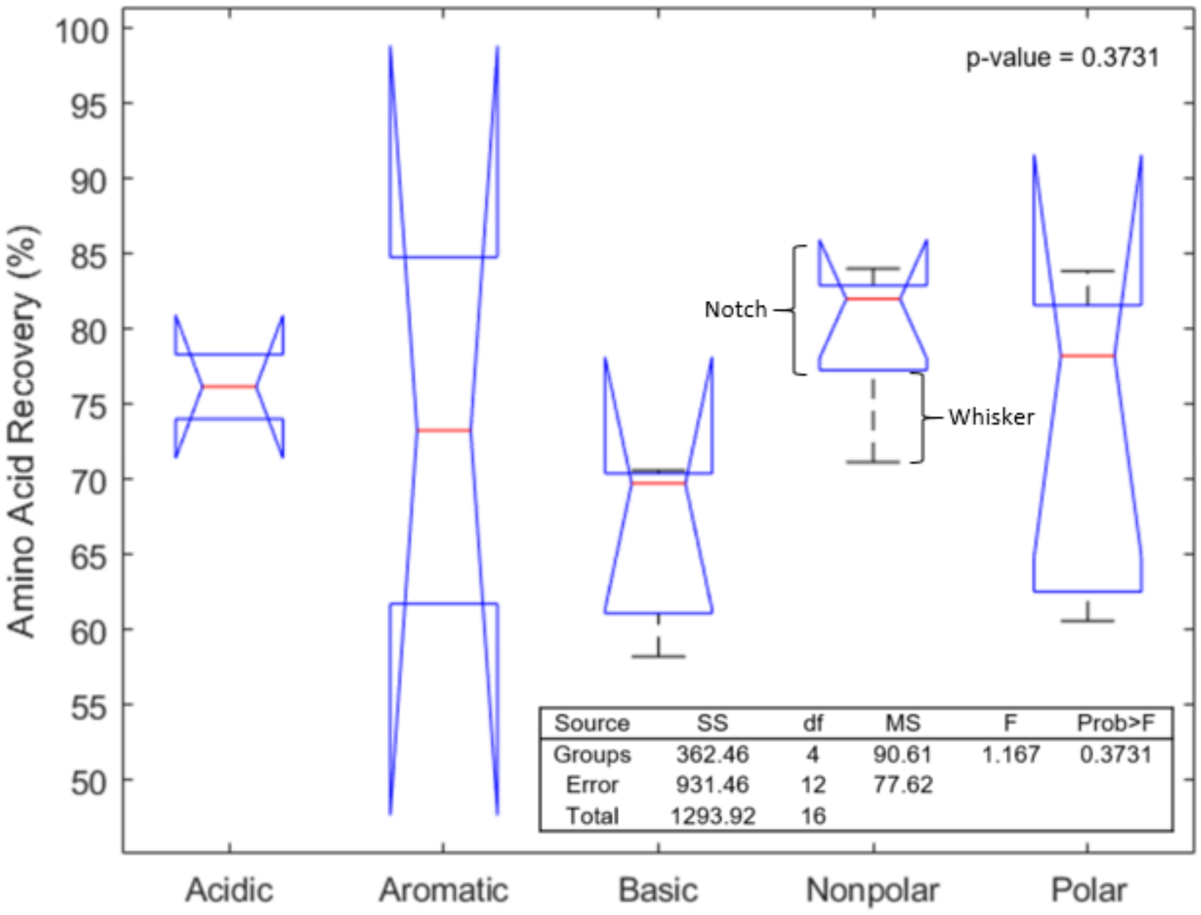

A one-way ANOVA analysis using the MATLAB anova1 function of the amino acid recoveries per type shows that there is no statistically significant difference between the five groups (p-value 0.3731). The red lines indicate the median for each dataset, the "notch" indicates the 95% confidence interval of the median, the blue horizontal lines indicate the 25th (bottom, first quartile) and 75th percentile (top, third quartile), and black "whiskers" indicate the maximum and minimum values of the dataset. Larger notches correspond to less certainty in the median, usually due to smaller sample sizes. Datasets without whiskers only had two amino acids in each group. The output table shows the sum of squares (SS), degrees of freedom (df), and the mean squared error (MS) for the between group variation (groups row), and the within group variation (error row). The calculated F-statistic is a ratio of the mean squared in the group to the mean squared in the error, and used to set the null hypothesis value. To be statistically significant, the test value must be greater than the F-statistic. The p-value is the probability this null hypothesis is true (Prob>F), and values less than 0.05 (significance value of 5%) reject the null hypothesis and indicate statistical significance.

Figure S5. Background contamination from resin in Figure 3

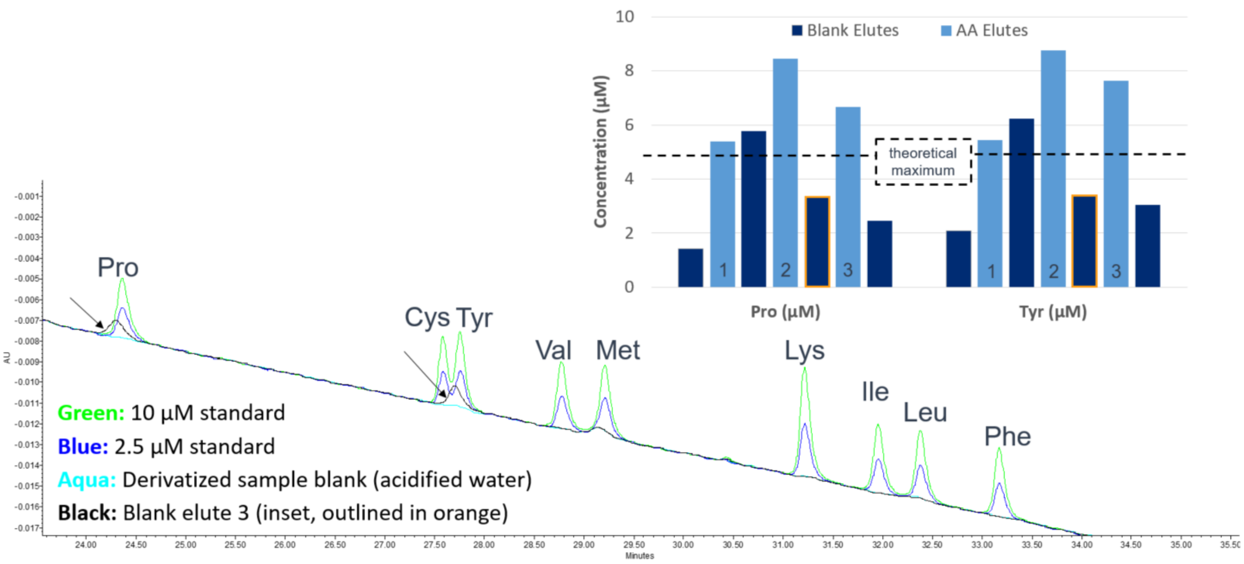

The above figure shows an example chromatogram for a blank run (black line) and ocean world analog separation (aqua line). Standard curves quantified the results, of which the 10 µm (green line) and 2.5 µm (blue line) are shown. For both proline and tyrosine, an unknown contaminant from the resin caused artificially high readings during the actual runs. This can be seen in the difference between the derivatized sample blank (aqua), and the blank elute 3 (black). The inset figure shows the levels of this contaminant compared to the theoretical maximum, calculated based on sample introduced. A slight spike is also seen under the met peak for both the derivatized sample blank and blank elute 3 that might have contributed to experimental uncertainty. The blank elute 3 that is shown in the chromatogram is outlined in orange. To determine actual concentration, we subtracted the value of the previous blank run (dark blue) from the actual run (light blue). Numbers on the bar indicate the three experiments run. In future, to both mitigate bead contamination issues and evaluate longevity for a deep space mission, we are investigating both UV-irradiation and thermal bake-out sterilization methods. Additionally, this resin was only certified to contain less than 100 microorganisms per gram as purchased, but we have the capability to synthesize our own in a clean room to minimize contamination.

Figure S6. ANOVA analysis of 100 µM ocean world analog experiment

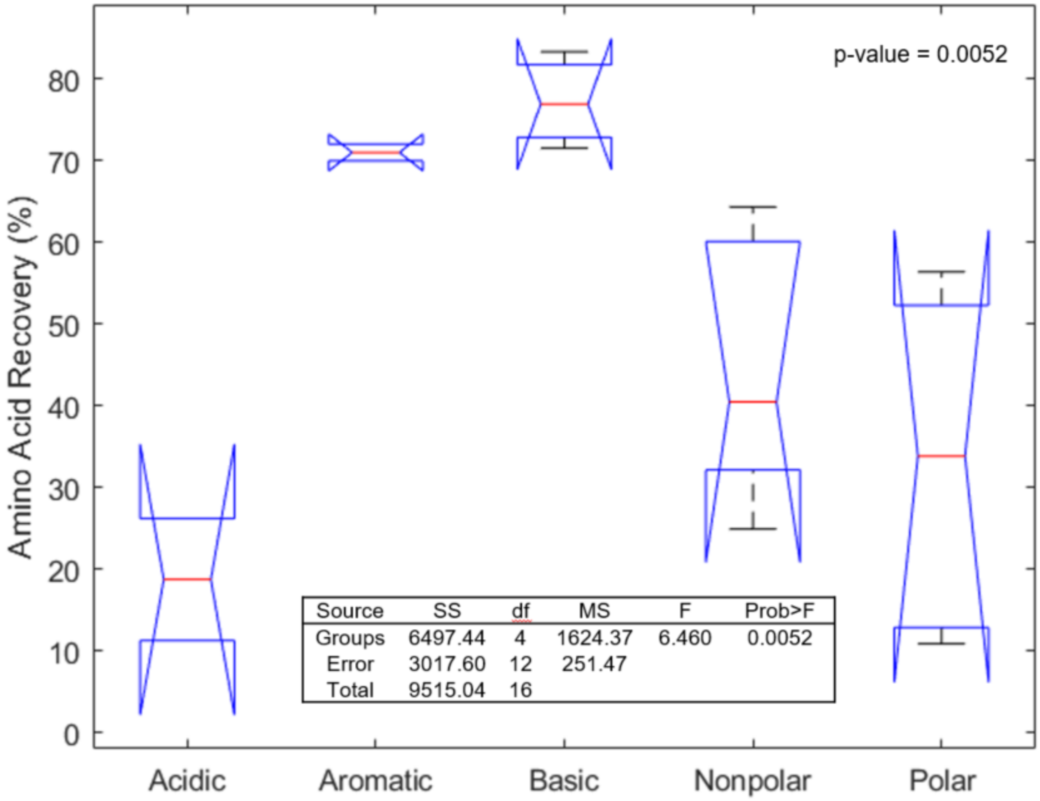

A one-way ANOVA analysis using the MATLAB anova1 function of the amino acids shows that the five groups are statistically significant (p-value 0.0052). Using a multiple comparison test (MATLAB function multcompare), it was determined that: 1) the mean in the acidic group is statistically significant from both the aromatic and basic groups, 2) the mean in the aromatic group is statistically significant from the acidic group, 3) the mean in the basic group is statistically significant from the acidic and polar groups, 4) the mean in the nonpolar group is not statistically significant from any group, and 5) the mean in the polar group is statistically different from the basic group.

Figure S7. Variability of experiments in Figure 5

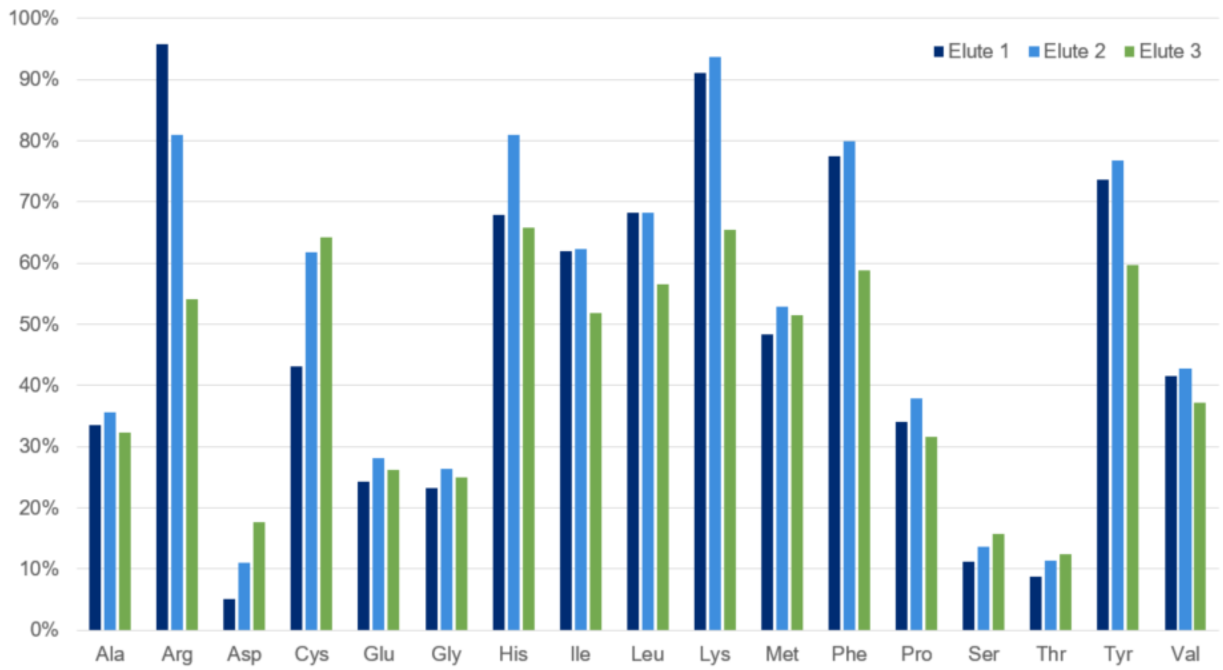

This figure shows the recovery difference in each of the seventeen amino acids between triplicate runs. In elute 3 there are lower recoveries than in elutes 1 and 2 for the three highest recovery amino acids (phe, lys, arg), and increases in recovery for the lower ones (asp, thr, ser). Because lower affinity cations are outcompeting higher affinity ones, we believe variability in sample loading volume due to syringe errors or small leaks may have caused this phenomena. Future design iterations will use automated fluid handling to reduce this.

Figure S8. ANOVA analysis of the enrichment experiment

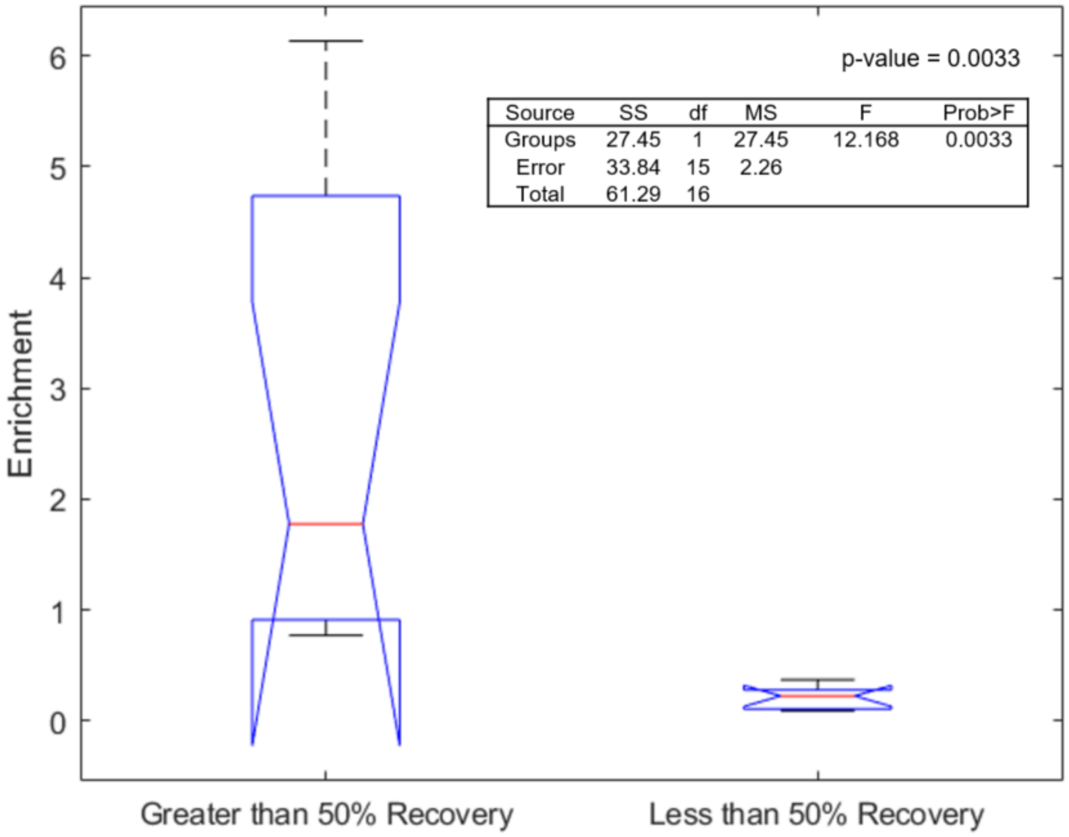

A one-way ANOVA analysis using the MATLAB anova1 function shows that the amino acids that had a greater than 50% recovery in **Figure 4** are statistically more likely to enrich than those that exhibited less than a 50% recovery (p-value 0.0033). This demonstrates the importance of choosing appropriate resin volumes when competition by both salts and amino acids are present.

Table S10. Theoretical resin bed calculations for amino acid retention

The table below calculates the theoretical resin bed sizes needed to retain all 17 amino acids in the ocean analog sample. All calculations assume a 1 mL total sample introduced, 1.7 mEq/mL available binding sites, and salt concentrations from ICP-MS results (**Table S7**). Case 1: Idealized scenario for total binding sites needed when there is no competition between amino acids or salts; Case 2: Amino acid binding affinity is from **Figure 4** (11 - 83%) and salt ion binding affinity is conservative at 80%; Case 3: Salts bind at 80% and the concentration of amino acids is very low (1 nM) with a very low affinity percentage (1%). The resulting bead bed sizes needed are only about 5x greater than the system we tested with, and would scale linearly with the volume of sample introduced."

Parameter	Case 1	Case 2	Case 3
Conc. of 17 AA Mix (µM)	100	100	.001
Amino Acid Affinity %	100%	11 – 83 % (Fig. 4)	1%
Salt Binding %	0	80%	80%
Total binding sites needed (mEq)	0.37	0.47	0.46

Resin Volume (μL)	218.6	275.3	272.0

References

Amashukeli X, Pelletier CC, Kirby JP, et al. Subcritical Water Extraction of Amino Acids from Atacama Desert Soils. J Geophys Res Biogeosciences 2007;112(G4); doi: 10.1029/2006JG000308.

Bada JL. A Search for Endogenous Amino Acids in Martian Meteorite ALH84001. Science 1998;279(5349):362–365; doi: 10.1126/science.279.5349.362.

Botta O, Martins Z and Ehrenfreund P. Amino Acids in Antarctic CM1 Meteorites and Their Relationship to Other Carbonaceous Chondrites. Meteorit Planet Sci 2007;42(1):81–92; doi: 10.1111/j.1945-5100.2007.tb00219.x.

Coury L. Conductance Measurements Part 1: Theory. Curr Sep 1999;6.

Glavin DP, Callahan MP, Dworkin JP, et al. The Effects of Parent Body Processes on Amino Acids in Carbonaceous Chondrites: Amino Acids in Carbonaceous Chondrites. Meteorit Planet Sci 2010;45(12):1948–1972; doi: 10.1111/j.1945-5100.2010.01132.x.

Glavin DP, Dworkin JP, Aubrey A, et al. Amino Acid Analyses of Antarctic CM2 Meteorites Using Liquid Chromatography-Time of Flight-Mass Spectrometry. Meteorit Planet Sci 2006;41(6):889–902; doi: 10.1111/j.1945-5100.2006.tb00493.x.

Liu D-L, Beegle LW and Kanik I. Analysis of Underivatized Amino Acids in Geological Samples Using Ion-Pairing Liquid Chromatography and Electrospray Tandem Mass Spectrometry. Astrobiology 2008;8(2):229–241; doi: 10.1089/ast.2007.0176.

Martins Z, Alexander CMO, Orzechowska GE, et al. Indigenous Amino Acids in Primitive CR Meteorites. Meteorit Planet Sci 2007;42(12):2125–2136; doi: 10.1111/j.1945-5100.2007.tb01013.x.

Martins Z, Price MC, Goldman N, et al. Shock Synthesis of Amino Acids from Impacting Cometary and Icy Planet Surface Analogues. Nat Geosci 2013;6(12):1045–1049; doi: 10.1038/ngeo1930.

McDonald GD and Bada JL. A Search for Endogenous Amino Acids in the Martian Meteorite EETA79001. Geochim Cosmochim Acta 1995;59(6):1179–1184; doi: 10.1016/0016-7037(95)00033-V.

Pizzarello S and Cronin JR. Non-Racemic Amino Acids in the Murray and Murchison Meteorites. Geochim Cosmochim Acta 2000;64(2):329–338; doi: 10.1016/S0016-7037(99)00280-X.

Pizzarello S, Huang Y and Fuller M. The Carbon Isotopic Distribution of Murchison Amino Acids. Geochim Cosmochim Acta 2004;68(23):4963–4969; doi: 10.1016/j.gca.2004.05.024.

Skelley AM, Scherer JR, Aubrey AD, et al. Development and Evaluation of a Microdevice for Amino Acid Biomarker Detection and Analysis on Mars. Proc Natl Acad Sci 2005;102(4):1041–1046; doi: 10.1073/pnas.0406798102.

Takano Y, Kashiyama Y, Ogawa NO, et al. Isolation and Desalting with Cation-Exchange Chromatography for Compound-Specific Nitrogen Isotope Analysis of Amino Acids: Application to Biogeochemical Samples. Rapid Commun Mass Spectrom 2010;24(16):2317–2323; doi: 10.1002/rcm.4651.

Takano Y, Sato R, Kaneko T, et al. Biological Origin for Amino Acids in a Deep Subterranean Hydrothermal Vent, Toyoha Mine, Hokkaido, Japan. Org Geochem 2003;34(11):1491–1496; doi: 10.1016/S0146-6380(03)00175-X.

Worstell J. Chapter 4 - Scaling Fluid Flow. In: Scaling Chemical Processes. (Worstell J. ed) Butterworth-Heinemann; 2016; pp. 77–107; doi: 10.1016/B978-0-12-804635-7.00004-0.

Zhao M and Bada JL. Determination of A-Dialkylamino Acids and Their Enantiomers in Geological Samples by High-Performance Liquid Chromatography after Derivatization with a Chiral Adduct of o-Phthaldialdehyde. J Chromatogr A 1995;9.
